# Exploring the Pattern of Immunization Dropout among Children in India: A District-Level Comparative Analysis

**DOI:** 10.3390/vaccines11040836

**Published:** 2023-04-13

**Authors:** Pritu Dhalaria, Sanjay Kapur, Ajeet Kumar Singh, Pretty Priyadarshini, Mili Dutta, Himanshu Arora, Gunjan Taneja

**Affiliations:** 1 Immunization Technical Support Unit, Ministry of Health & Family Welfare, New Delhi 110070, India; pritu_dhalaria@in.jsi.com (P.D.); pretty_priyadarshini@in.jsi.com (P.P.); mili_dutta@in.jsi.com (M.D.); 2 John Snow India, New Delhi 110070, India; sanjay_kapur@in.jsi.com; 3 Kantar Public, New Delhi 110019, India; himanshu.arora@kantar.com; 4 Bill & Melinda Gates Foundation, New Delhi 110067, India; gunjan.taneja@gatesfoundation.org

**Keywords:** dropout rate, immunization, indicator, health, sustainable development goal

## Abstract

The dropout rate is one of the determinants of immunization coverage and program performance, program continuity, and follow-up. The dropout rate refers to the proportion of vaccine recipients who did not finish their vaccination schedules, and it is determined by comparing the number of infants who started the schedule to the number who completed it. It is the rate difference between the first and final dosage or the rate difference between the first vaccination and the last vaccine dropout; thus, it denotes that the first recommended dose of vaccine was received, but that the subsequently recommended dose was missed. In India, immunization coverage has shown significant improvements over the last two decades, but full immunization coverage has remained stagnant at 76.5%, of which 19.9% are partially immunized, and 3.6% are children who have been left out. In India, the Universal Immunization Programme (UIP) is challenged with cases related to dropout in immunization. Although immunization coverage in India is improving, the program is challenged by vaccination dropouts. This study provides an analysis of the determinants of vaccination dropout in India using data from two rounds of the National Family Health Survey. The finding shows that the mother’s age, education, family wealth, antenatal care visit, and place of delivery were some of the variables that significantly contributed to reducing the dropout rate of immunization among children. The findings of this paper show that the dropout rate has reduced over a certain period of time. The overall improvement in the rates of dropout and increase in full immunization coverage could be attributed to various policy measures taken in the last decade in India, which brought structural changes with a positive impact on full immunization coverage and its components.

## 1. Introduction

Immunization is a process whereby an individual becomes immune or resistant to an infectious disease, typically by the administration of a vaccine. Childhood immunization is a preventive health behavior that is primarily taken care of by parents and caregivers [1]. It has been proven as one of the most cost-effective health interventions worldwide, through which several childhood diseases have been prevented or eradicated [2]. The Expanded Program on Immunization (EPI) was launched by the World Health Organization (WHO) in 1974, and since then, vaccines have significantly reduced vaccine-preventable diseases (VPDs) and deaths worldwide [3]. The administration of each vaccine dose ensures that each child is immunized at the end of their first year of life, as planned by the EPI. Immunization plays a critical role in achieving the Sustainable Development Goals (SDGs), specifically SDG 3— ‘Ensure healthy lives and promote well-being for all at all ages”—and also contributes directly or indirectly to 13 other SDGs. Childhood vaccination is one of the core strategies for reducing under-five mortality to fewer than 25/1000 live births by 2030 under SDG 3 [4].

Vaccination coverage is reducing in many developing countries; even where good coverage has been attained, it has been difficult to reach out to unvaccinated children [5]. Immunization Agenda 2030 (IA2030) aims to vaccinate everyone by increasing equitable access to and full utilization of existing and new vaccines [3]. The benefits of immunization are not uniform, and the coverage varies widely among and within countries. Moreover, there is limited access to vaccination programs in unstable, migratory, and conflict-torn contexts, which comprise the poorest, marginalized, and most vulnerable populations [6]. Each year, 20 million infants do not receive a full course of even basic vaccines, and many more miss out on newer vaccines [7].

Despite worldwide gains in immunization, underdeveloped nations have faced challenges in utilizing childhood vaccination programs. Children are immunized cost-free through a variety of routine, scheduled, and outreach vaccination programs [8]. The 2018 Global Vaccine Action Plan (GVAP) assessment report did, however, reveal that despite a global trend of increasing immunization coverage rates, the achievement of these immunization coverage indicators lagged behind, especially in low- and middle-income countries [2]. The WHO recommends that complete immunization coverage should reach at least 90% of children at the country level and 80% at the district level [9]. Immunization systems’ performance is measured via multiple dimensions, including immunization coverage, immunization dropout, equity of coverage, the completeness of vaccines in the national immunization schedule compared with recommended vaccines, and other administrative indicators [10].

The dropout rate is one of the determinants of immunization coverage and program performance, program continuity, and follow-up. Immunization dropout signifies that the child has received the first recommended dose of the vaccine, yet has missed the next recommended dose. It is the rate difference between the first and final dosage or the rate difference between the first vaccination and the last vaccine dropout, so it denotes that the first recommended dose of the vaccine was received, but the subsequent recommended dose was missed [11,12]. The dropout rate indicates whether there is an accessibility issue, i.e., whether there is difficulty in reaching out to the immunization services for subsequent doses or there is an issue with the utilization of the services [13]. Reducing immunization dropout rates is crucial for achieving high full immunization coverage rates. The immunization dropout rate reduces the effectiveness of immunization programs, as even a small percentage of children who fail to complete their vaccinations can significantly decrease the overall full immunization rates.

Low dropout rates are critical to preventing morbidity and mortality from VPDs. As per the WHO and CDC, a dropout rate greater than 5% is an indicator of immunization program performance; on the other hand, a dropout rate greater than 10% is unacceptable, and a dropout rate of >10% reflects the underutilization of immunization services [9,14]. The DPT1-MCV1 is preferred, as it can measure dropout over a longer time interval between doses. In addition, the DPT1–DPT3 dropout rate measures the ability of the immunization system to reach a child multiple times with the same antigen(s) [15,16,17]. Closing the dropout ultimately leads to complete immunization. Several strategies were deployed in subsequent years to address the low immunization coverage, including RI strengthening, supplemental immunization activities, a global positioning system tracker, and several community-level interventions [18]. Vaccine uptake is one of the key performance indicators of immunization programs. Risk factors for incomplete vaccination may be different from the risk factors for non-vaccination [19].

Despite considerable progress and the introduction of an increasing number of vaccines into the EPI, many children continue to go unvaccinated by their first birthday or never complete the recommended schedule. Despite overall improvements in immunization coverage at the national level, geographic variations in immunization coverage persist at most subnational and district levels in India [20]. Although immunization coverage has shown significant improvements over the last two decades, full immunization coverage has remained stagnant at 76.5%, of which 20% are partially immunized, and 3.6% are children who have been left out [21]. In India, the Universal Immunization Programme (UIP) is challenged with cases related to dropout in immunization. Due to its large population, the country still has a significant number of partially vaccinated children, and there are huge variations in immunization coverage across regions [22]. The distribution of immunization coverage, timeliness, and dropout rate have been heterogeneous across health districts due to inadequacy in immunization demand and/or service delivery. A global review of the grey literature by Favin et al. found that the prime reasons for incomplete vaccination were unpleasant experiences at the immunization center (e.g., poor treatment of child caregivers at the health centers, long waiting time, lack of availability of drugs), missed opportunities (e.g., health workers’ refusal to immunize sick children, turning away a child who lacked a vaccination card, lack of availability of the vaccine), fear of side effects, and inadequate knowledge about the vaccination schedule [5].

In 2019, an estimated 14 million children were unimmunized and 5.7 million were under-immunized; these were predominantly children from lower socioeconomic classes and rural populations in LMICs [23].

At present, there is 93.6% coverage for DPT1 and 86.7% coverage for DPT3 in India. A handful of studies have investigated the reasons behind dropout after the first dose of DPT or oral polio vaccine (OPV) globally, albeit within a very limited scope [24,25,26,27]. Therefore, there is a need to explore the factors contributing to the dropout cases from vaccination in India from the time of first vaccine is scheduled (BCG) to the last vaccine in the schedule (MCV1) which is given in the first year of life. Therefore, the study also aimed to examine the antigen-wise dropout and various determinants of change in immunization dropout between two points in time. The overarching aim of the present study was to examine the immunization dropout rate and equity gaps among children aged 12–23 months in India.

## 2. Method and Materials

### 2.1. Data Source

**Figure 1:**
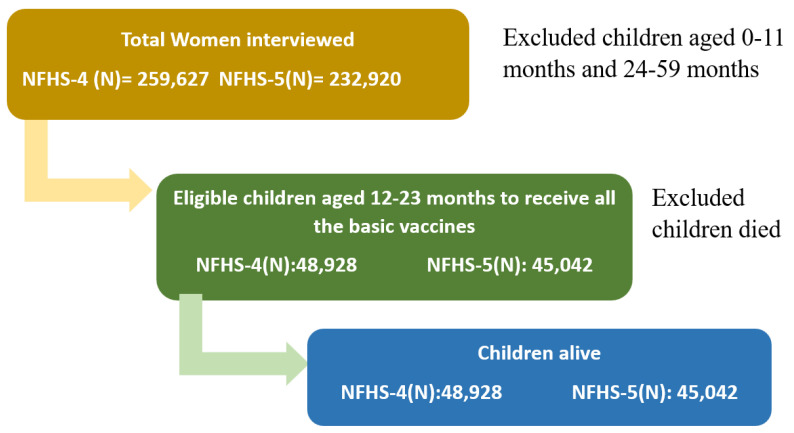
Selection procedure of the study sample from NFHS-4 and NFHS-5.

This study was based on secondary data sources. We used data from the fourth and fifth rounds of the National Family Health Survey (NFHS-4, NFHS-5) [21,28]. The NFHS surveys are nationally representative cross-sectional surveys that provide essential information on health and family welfare, maternal and child health, and reproductive health indicators. NFHS surveys are conducted by the Ministry of Health and Family Welfare (MoHFW). NFHS-4 was conducted in 2015–2016, and the most recent NFHS-5 was conducted in 2019–2021. Both surveys provide district-level estimates. A total of 699,686 women from 601,509 households were interviewed in the NFHS-4 survey, whereas 724,115 women from 636,699 households were interviewed in the NFHS-5. Among them, 259,627 and 232,920 women in NFHS-4 and 5, respectively, had at least one child ever born, and they were chosen for information on their children. In our study, we selected children from 12–23 months of age. The selection criteria of the sample are illustrated in Figure 1.

### 2.2. Immunization Services in India

The immunization program in India was launched in 1978 as part of the EPI. Later in 1985, it was renamed the Universal Immunization Programme. The UIP is targeting nearly 26.7 million newborns and 29 million pregnant women annually [20,29]. Table 1 outlines the immunization schedule in India. The Pentavalent vaccine was introduced in 2011 as a replacement for the DPT and Hepatitis B vaccines. It contains five antigens, namely, the Hepatitis B, Diphtheria + Pertussis + Tetanus (DPT vaccine), and Hemophilus Influenza b (Hib). The Rotavirus vaccine was launched in 2016 in a phased manner. The NFHS-4 survey did not provide information on the Pentavalent or the Rotavirus vaccines; however, it contained information on the DPT vaccine. Therefore, for the current study, data on DPT vaccines from NFHS-4 and DPT/Penta vaccines from NFHS-5 were considered for comparison purposes.

### 2.3. Dependent Variable

The immunization dropout rate was considered as the dependent variable, indicating that the first recommended dose of the vaccine was received and the next recommended dose was missed. The current study analyzed the dropout rate of BCG to MCV1, OPV1 to OPV3, and DPT/Penta1 to DPT/Penta3. The dropout rate was calculated by dividing the number of children aged 12–23 months who received the first recommended dose minus the children aged 12–23 months divided by the number of children aged 12–23 months who received the first dose. The following dropout rates were used in the present study:

(1) $$Dropout\;Rate\;BCG\;to\;MCV1=\frac{BCG-MCV1}{BCG}\times 100$$

(2) $$Dropout\;Rate\;OPV1\;to\;OPV3=\frac{OPV1-OPV3}{OPV1}\times 100$$

(3) $$Dropout\;Rate\;DPT1\;to\;DPT3=\frac{DPT1-DPT3}{DPT1}\times 100$$

### 2.4. Independent Variables

After the literature review, the following variables were considered as predictor variables—mother’s age (15–19 years, 20–29 years, or 30 and above years), place of residence (urban or rural), mother’s education level (no education, primary education, secondary education, or higher education), religion (Hindu, Muslim, or others), wealth index (poorest, poorer, middle richer, or richest), birth order (first, second, third, fourth, or above), sex of the child (male or female), antenatal visits (no visit, 1–3 visits, or 4 and above visits), place of delivery (home or public health facilities), and place of vaccination (public health facilities or private health facilities). The wealth index was generated using principal component analysis from data on household ownership of selected assets.

### 2.5. Statistical Analysis

A descriptive analysis was conducted of the coverage of antigens, including BCG, OPV-1, OPV-2, OPV-3, DPT-1, DPT-2, DPT-3, MCV-1, and full immunization. Dropout rates were estimated for both NFHS-4 and NFHS-5 surveys. A bivariable analysis of dropout rates by selected background characteristics was conducted. Furthermore, logistic regression was used to understand the factors affecting dropout rates by background characteristics. The adjusted odds ratios, *p* values, and confidence intervals (CI) at 95% were estimated and shown. In order to decompose the gap in dropout rates in NFHS-4 and NFHS-5, we employed the Fairlie decomposition method, which is suitable for binary outcomes. The Fairlie decomposition method is an extension of the Oaxaca–Blinder decomposition method. Oaxaca–Blinder decomposition is used for continuous variables and uses linear regression models. Therefore, in our study, due to the dichotomous nature of the outcome variable, we used the Fairlie decomposition method. The methodology is briefly described below:

The decomposition for a non-linear equation, y = F(xβ), can be written as

(4) $${\bar{y}}^{o}-{\bar{y}}^{s}=\left[\sum_{i=1}^{N^{o}}\frac{F\left(x_{i}^{o}\beta^{o}\right)}{N^{o}}-\sum_{i=1}^{N^{s}}\frac{F\left(x_{i}^{s}\beta^{o}\right)}{N^{s}}\right]+\left[\sum_{i=1}^{N^{o}}\frac{F\left(x_{i}^{s}\beta^{o}\right)}{N^{s}}-\sum_{i=1}^{N^{s}}\frac{F\left(x_{i}^{s}\beta^{s}\right)}{N^{s}}\right]$$

where N*^j^* is the sample size for interest group *j*, y^j^ is the average probability of the binary outcome of the interest group *j,* and F is the cumulative distribution function from the logistic distribution. The above equation implies the observed change between NFHS rounds from group differences in the distribution of observable characteristics (explanatory variable effects) and group differences in unobservable characteristics (coefficient effects). The first term in brackets in the above equation represents the part of the gap between the above measures due to group differences in distributions of the entire set of independent variables or observed characteristics, and the second term represents the part due to differences in the group processes which determined the levels of y (coefficient effect). The second term also captures the portion of the group gap due to group differences in immeasurable or unobserved endowments.

## 3. Results

### 3.1. Study Characteristics of the Sample

Table 2 shows that overall, 48,928 samples from NFHS-4 and 45,042 samples from NFHS-5 were analyzed. The sample characteristics are also shown in Table 2. In the sample, the majority of mothers’ ages fell into the 20–29 age group category, came from rural regions, completed a secondary education, belonged to the Hindu religion, and were in the poorest wealth quintile. The sample consisted of children of the first and second birth orders. The place of delivery and place of vaccination were majorly public health facilities.

### 3.2. Antigen-Wise Coverage and Dropout Rates

**Figure 2:**
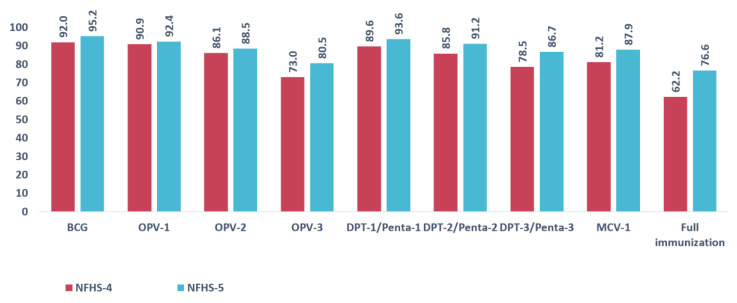
Antigen wise coverage percentage in NFHS-4 and NFHS-5.

Overall, the full immunization coverage was 62.6% in NFHS-4 and 76.6% in NFHS-5, which reflects an increase of 14 percentage points in full immunization between these two surveys. The highest coverage was observed for the BCG vaccine in both NFHS-4 (92.0%) and NFHS-5 (95.2%). The results show that the proportion of coverage of antigens increased from the time of the NFHS-4 survey to that of NFHS-5. A major increase of 8.2% was found in DPT-3 vaccine coverage was found in NFHS-4. For OPV-3 and MCV-1, increases of 7.5% and 6.7% in coverage, respectively, were observed in NFHS-5 (Figure 2).

**Figure 3:**
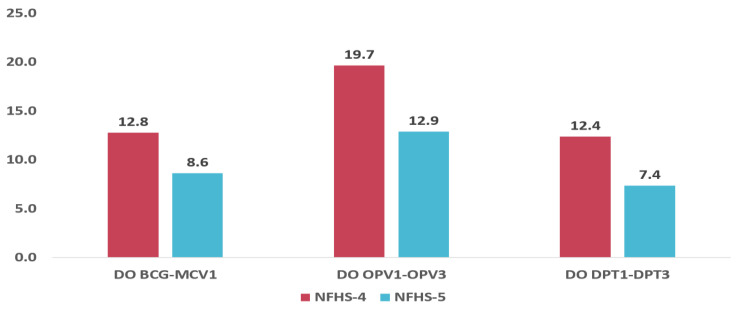
Antigen wise dropout rate in NFHS-4 and NFHS-5.

Figure 3 shows the proportion of children who received the first recommended dose and dropped out before receiving the scheduled second dose. The dropout rates of BCG to MCV1, OPV1 to OPV3, and DPT1 to DPT3 were estimated. In NFHS-4, the dropout rate for BCG-MCV1 was 12.8%; for OPV1-OPV3, 19.7%; and for DPT-1 to DPT-3, 12.4%. Figure 3 highlights that the highest dropout was observed between OPV1 to OPV3 as compared to the other two scenarios. The dropout rate decreased considerably in the NFHS-5 survey. The dropout rates were 8.6%, 12.9%, and 7.4% for BCG-MCV1, OPV1-OPV3, and DPT1-DPT3, respectively, in NFHS-5.

### 3.3. State-Wise Dropout Rates

**Figure 4:**
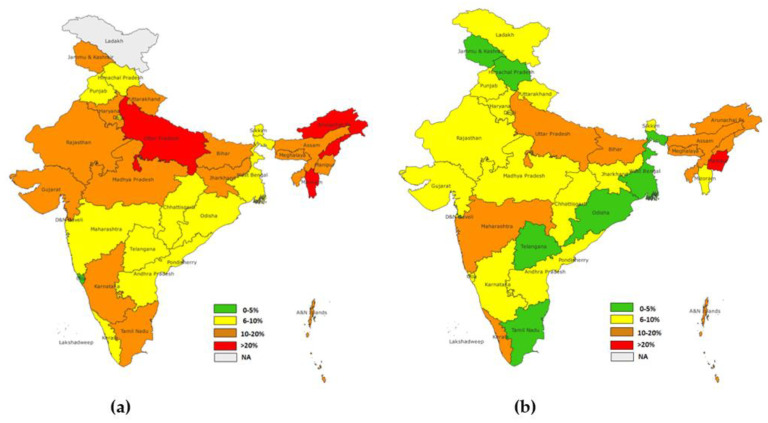
(**a**) State-level dropout rate for BCG-MCV1(NFHS-4), (**b**) state-level dropout rate for BCG MCV1(NFHS-5).

Further, state-wise dropout rates for NFHS-4 and NFHS-5 were estimated for the states of India. The dropout rates for BCG-MCV1 were the highest in Uttar Pradesh (20.4%), according to NFHS-4, and in Assam (12.0%), according to NFHS-5. A considerable decrease in the dropout rates of BCG-MCV1 was observed in the majority of the states. However, Kerala, Maharashtra, Chhattisgarh, and Punjab observed increased dropout rates. (Figure 4).

**Figure 5:**
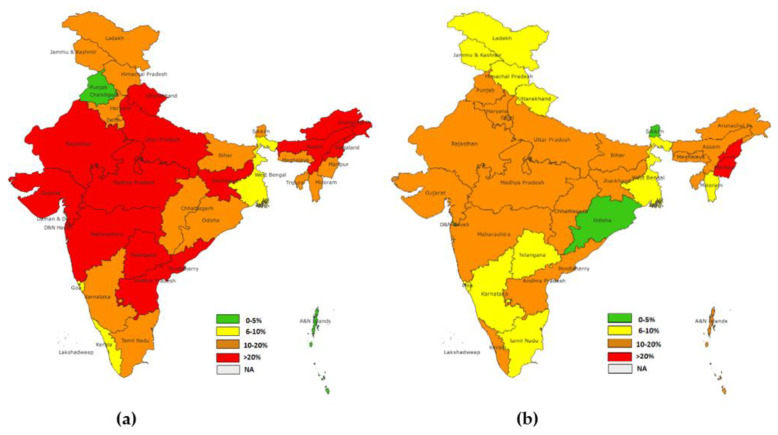
(**a**) State level dropout rate for OPV1-OPV3 (NFHS-4), (**b**) state level dropout rate for OPV1-OPV3 (NFHS-5).

The state-wise dropout rate of OPV1-OPV3 showed the highest dropout in NFHS-4 in Dadra and Nagra Haveli (37.6%), followed by Assam (32.4%). However, in NFHS-5, the highest dropout was in Nagaland (23.8%), followed by Manipur (20.7%). A significant reduction in the dropout rate was observed in the majority of the states, while states such as Kerala and Punjab showed an increase in the dropout rates (Figure 5).

**Figure 6:**
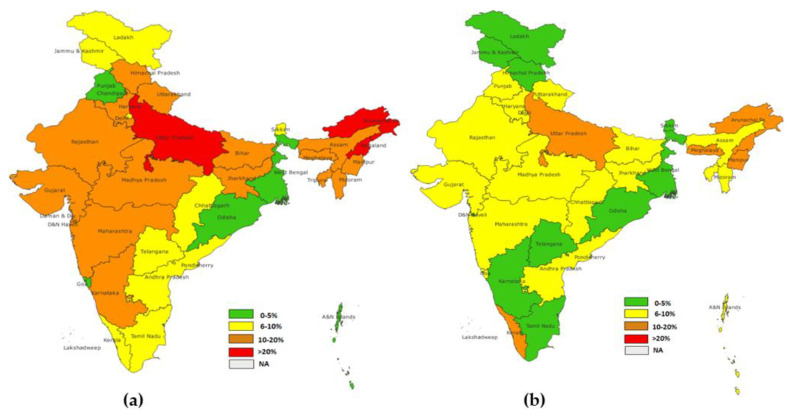
(**a**) District level dropout rate for DPT1-DPT3 (NFHS-4), (**b**) district level dropout rate for DPT1-DPT3 (NFHS-5).

The highest dropout rate for DPT1-DPT3 of the NFHS-4 group was observed in Nagaland (23.9%), followed by Arunachal Pradesh (23.7%) and Uttar Pradesh (20.4%). In the NFHS-5 group, the highest dropout rate was observed in Nagaland (15.0%) and Manipur (12.9%), followed by Kerala (11.0%) and Uttar Pradesh (11.05%) Similarly to other dropout rates which were considered, (BCG-MCV1, OPV1-OPV3) the majority of states showed a reduction in rates, except for Kerala, Punjab, and Chhattisgarh, where an increase in dropout rates was observed (Figure 6).

### 3.4. District-Level Dropout Rates in NFHS-4 and NFHS-5

**Figure 7:**
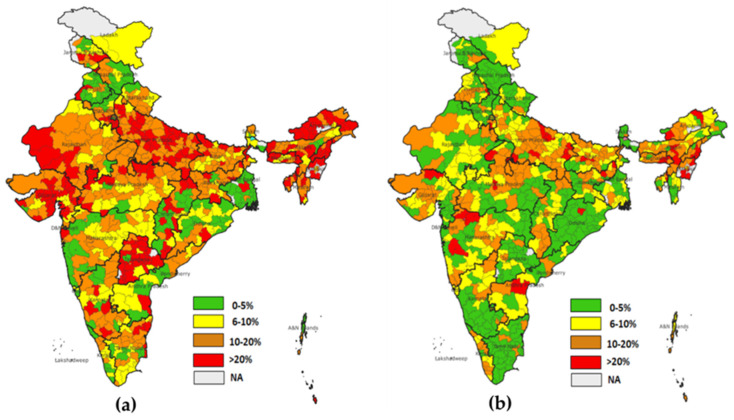
(**a**) District level dropout rate for BCG-MCV1 (NFHS-4), (**b**) district level dropout rate for BCG-MCV1(NFHS-5).

The maps for district-level dropout rates clearly show that dropout rates have decreased considerably across districts from 2015–2016 to 2019–2021. A higher dropout for BCG-MCV1 was found in the majority of northeastern districts, including Nagaland, Mizoram, Tripura, Manipur, Meghalaya, Uttar Pradesh, and Gujarat. In NFHS-4, nearly 124 districts had dropout rates of more than 20%, while only 37 districts had dropout rates of more than 20% in NFHS-5. This indicates that substantial improvement in the dropout rate for BCG-MCV1 occurred in 5 years (Figure 7).

**Figure 8:**
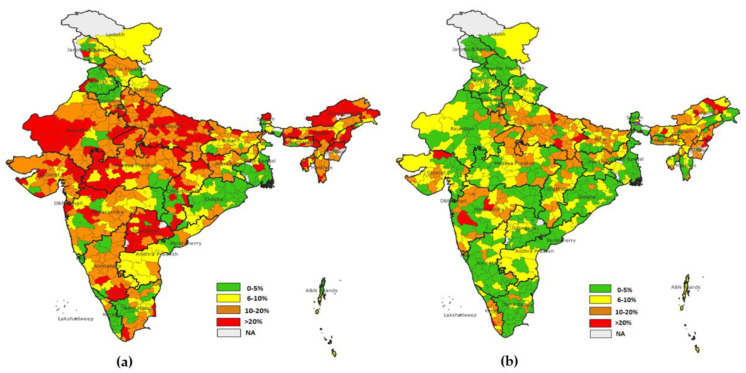
(**a**) District level dropout rate for DPT1-DPT3 (NFHS-4), (**b**) district level dropout rate for DPT1-DPT3 (NFHS-5).

DPT-3 shows that in NFHS-4, 126 districts had dropout rates of more than 20%, while in NFHS-5, only 17 districts had dropout rates of more than 20% (Figure 8).

**Figure 9:**
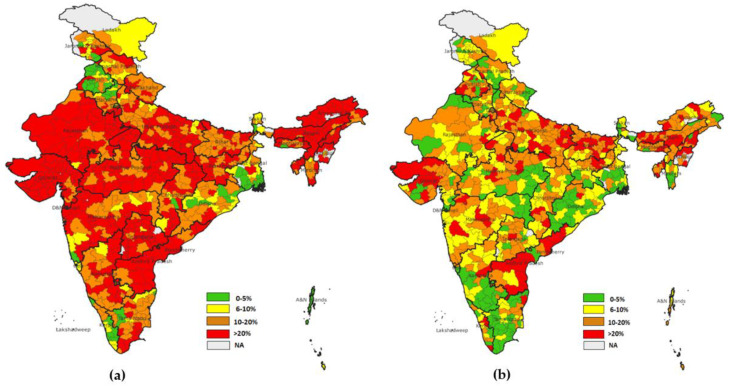
(**a**) District level dropout rate for OPV1-OPV3(NFHS-4), (**b**) district level dropout rate for OPV1-OPV3 (NFHS-5).

The dropout rate for OPV1-OPV3 was the highest observed across the districts of India. It was found that in NFHS-4, 299 districts had more than 20% dropout rates, and in NFHS-5, this was true for 115 districts. The highest dropout rates were observed in Nagaland, Manipur, Meghalaya, Bihar, and Andhra Pradesh (Figure 9).

### 3.5. Dropout Rates by Background Characteristics

The dropout rates were higher in children whose mothers were not educated. It was observed that as the level of education of the mother increased, the dropout rates decreased. The dropout rate was also higher among mothers belonging to the poorest quintile. The dropout rate decreased considerably as the standard of living increased. It was evident that there were lower dropout rates among first-order births, while for higher birth orders, the level of dropout increased. Similarly, dropout rates were higher in children delivered in the home, as mothers did not receive any antenatal care, and vaccination was performed in private health facilities (Table 3).

### 3.6. Factors Affecting Dropout Rates

Table 4 shows predictors of immunization dropout in the study area. In the binary multivariate analysis, it was found that mothers aged 20–29 years (OR: 0.805, 95% CI: 0.69–0.94) and 30 and above years (OR: 0.728, 95% CI:0.61–0.86) were 20% and 27% less likely to drop out from BCG-MCV1 immunization schedule as compared to mothers aged 15–19 years in 2015–2016, and 44% and 58% less likely in 2019–2021. A similar relation of the mother’s age with the dropout rate of OPV1-OPV3 and the dropout rate of DPT1-DPT3 was observed across surveys. Rural women, as compared to urban women, were less likely to drop out of the immunization schedule. The mother’s education level was found to be a significant factor in reducing the dropout rates. It was observed that as the level of education increased among mothers, the dropout rate decreased. Mothers who completed higher education were 44%, 26%, and 23% less likely to drop out of BCG-MCV1, OPV1-OPV3, and DPT1-DPT3 immunization schedules, respectively, in 2019–2021. Children of Muslim mothers and mothers from other religions were more likely to drop out as compared to children of Hindu mothers. The mother’s wealth index status was found to significantly affect the dropout rates. It was observed that as the wealth index status of mothers improved from poorer to richer, the likelihood of dropping out of the immunization schedule decreased considerably. This relationship was the same for all vaccination dropout schedules considered in the study across different surveys.

Third and subsequent birth order children were found to be more likely to miss the vaccination schedule as compared to children of first-order birth. Antenatal visits were also found to be a major contributing factor to decreasing the dropout rate. Mothers who attended 4 or above antenatal visits were less likely to drop out of the immunization schedule. It was observed that women who attended 4 or above antenatal visits were 45%, 38%, and 40% less likely to drop out of BCG-MCV1, OPV1-OPV3, and DPT1-DPT3, respectively. Place of delivery and place of vaccination were also found to be associated with dropout rates. It was observed that women who delivered in public and private hospitals were less likely to drop out. It was also found that children vaccinated in private health facilities were more likely to drop out as compared to children vaccinated in public health facilities.

### 3.7. Decomposition Result of Gap in the Dropout Rate

Table 5 provides a summary of the decomposition analysis. The dropout rate probability values for BCG-MCV1 were 0.137 and 0.101 in NFHS-4 and NFHS-5, respectively. The dropout rate probability values for OPV1-OPV3 were 0.200 and 0.132 in NFHS-4 and NFHS-5, respectively. The dropout rate probability values for DPT1-DPT3 were 0.129 and 0.073 in NFHS-4 and NFHS-5, respectively. The gaps between the surveys for dropout rates were 0.036, 0.069, and 0.057 for BCG-MCV1, OPV1-OPV3, and DPT1-DPT3, respectively.

The results showed that about 56% of the gap in the dropout rate of BCG-MCV1 between the surveys could be explained by the predictors included in the analysis. However, the predictors explained nearly 37% and 39% of the gaps in the dropout rates of OPV1-OPV3 and DPT1 to DPT3, respectively, between the surveys.

**Figure 10:**
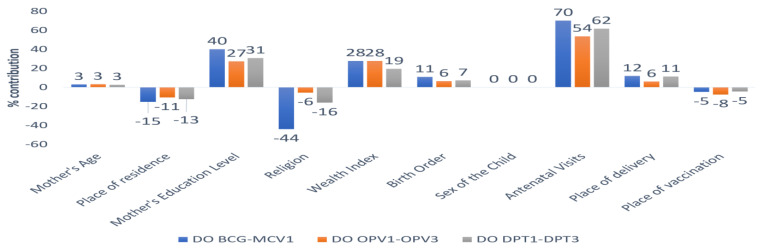
Percentage contribution of each covariate to the gap in dropout rates between the NFHS-4 and NFHS-5 surveys.

Figure 10 presents the details of the decomposition analysis, showing the contribution of predictors to explaining the gaps in dropout rates between the surveys. The results revealed that antenatal visits were the main contributor, explaining about 70% of the gap in BCG-MCV1 dropout between the surveys. Similarly, antenatal visits were a major contributor to the gaps in the dropout rates of OPV1-OPV3 and DPT1-DPT3 between the surveys. The mother’s level of education was another important contributor, explaining 40% of the gap in BCG-MCV1 dropout, 27% of the gap in OPV1-OPV3 dropout, and 31% of the gap in DPT1-DPT3 dropout. The third major contributor was the wealth index, explaining 28%, 28%, and 19%, respectively, for DO BCG-MCV1, DO OPV1-OPV3, and DO DPT1-DPT3. Place of delivery also explained 12%, 6%, and 11%, respectively, for the considered dropout rates. The sex of the child was found to have no effect on the gaps in dropout rates between the surveys.

## 4. Discussion

Vaccines have the power not only to save lives, but also to give children a chance to grow up healthy and improve their life prospects. Though immunization coverage in India is improving, the program is challenged by vaccination dropouts. However, immunization will become more effective if children receive the full course of recommended vaccination doses. The problem of dropout has different program-related consequences as compared to left-out children. Routinely, dropout is used as an indicator of an immunization program’s performance, and low dropout rates indicate good access to and utilization of immunization services. This study provides an analysis of the determinants of vaccination dropout in India using data from the National Family Health Survey. The findings show that the mother’s age, education, family wealth, antenatal care visits, and place of delivery were some of the variables that significantly contributed to reducing the dropout rate of immunization among children. There was inter-survey improvement, as the reduction in dropouts contributed to about a 14% increase in full childhood vaccination coverage [21,28]. The current study revealed that BCG has the highest coverage, followed by Penta-1. The findings also revealed a high dropout rate of 12.9% between OPV-1 and OPV-3 and our results are in line with a few other studies [15,29]. The state and district-level dropout rates show that among all the states, the dropout rates were high among the north-eastern states of India.

The findings show that children born from mothers who were less educated were more likely to drop out across all antigens as compared with those born from mothers who were more educated. This is because mothers who are well educated might have greater autonomy, shifts from conventional views, and control over household resources. As a result, they may have improved healthcare-seeking behavior and may be capable of absorbing new health information more rapidly. Similarly, the findings also demonstrated that children belonging to the poorest wealth quintile were more likely to drop out of vaccination as compared with children in the highest wealth quintile across all antigens. Caregivers may dwell in richer households; thus, they might not have barriers to accessing services at health facilities compared to poorer families. The findings of this paper also reflect that religion plays a significant role in explaining dropouts. The Muslim population was more likely to drop out of vaccination as compared to the non-minority population. The possible explanation for this difference might be due to inadequate information provision about the importance of child vaccination completion while receiving maternal and child health services from health personnel, in addition to socio-cultural differences and lack of trust [10,30]. This study also found that the age of the mother was significantly associated with dropout. Mothers aged 20 years or older were less likely to drop out than those aged 20 years or younger. This result was supported by findings of other studies conducted at the state level in India. This may be due to early pregnancy, lack of knowledge of antenatal and postnatal care, and poor home visit rounds by health workers for counselling young mothers [11]. This shows that the likelihood of defaulting from completion of child vaccination was greater among mothers/caregivers residing in urban areas across all antigens, in the wake of rapid urbanization in recent years. This study was consistent with studies conducted in other parts of the world that revealed a failure to cater to the urban poor and slum population, and this is considered as an obstacle to achieving complete vaccination [11,26,31,32,33]. A possible explanation of these findings may be the loss of access to health facilities in urban slums and the lack of advice regarding the benefits of child vaccination that causes mothers/caregivers to default from child immunization. The other explanation for rural areas’ high vaccination coverage is that frontline health workers in rural India serve as an important link to authorized health institutions by providing various services to people’s homes. Accredited social health activists (ASHA), Anganwadi (AWW), and auxiliary nurse midwives (ANM) are vital frontline health professionals who are recruited from the communities they serve. Rural communities are more tightly bonded than metropolitan populations. The multidimensional nature of the rural healthcare system makes involvements outside the conventional design essential for ensuring the smooth delivery of services.

The findings of our study are in line with the previous literature on immunization dropout in general. Vaccination status was significantly associated with the full completion of antenatal check-ups. Mothers who attended regular ANC follow-ups or completed 4 ANCs were 45% (BCG-MCV1), 38% (OPV1-OPV3), and 40% (DPT1-DPT3) less likely to drop out as compared to their counterparts [18,26,34]. A possible explanation could be that mothers who have not visited health facilities for ANCs might have no exposure to information about the importance of completing vaccination. Place of delivery was also associated with vaccination dropouts. Children delivered at a public health facility were more likely to complete the vaccination schedule as compared to children delivered at home-based facilities [35,36]. The findings of our study also demonstrated that dropout was more likely with child immunization in private health facilities as compared to public health facilities. In general, it has been observed that in private health facilities, immunization is more expensive. At public hospitals, the average cost of immunization is lower than in private hospitals. Whatever individuals spend on immunization in public hospitals may be ascribed to transportation costs or other indirect costs, as immunization in public hospitals is free in India. The high dropout rate for the OPV vaccine may be due to the difficulty of maintaining accurate records of oral vaccines such as OPV, as they are often administered in campaign mode outside of clinical settings. This can make it challenging to properly document the administration of the vaccine, unlike injectable vaccines, which are typically given in a health facility and recorded in the child’s immunization record. The overall improvement in dropout and increase in full immunization coverage could be attributed to various policy measures undertaken in the last decade in India, such as the electronic vaccine intelligence network, Misson Indhradhanush, Intensified Mission Indradhanush, and the Janani Suraksha Yojana (JSY) scheme, which have strengthened the public health system and brought about structural changes with a positive impact on full immunization coverage and its components. However, the study has a few limitations as well. The study was based on large-scale, nationally representative, household-based survey data. The information collected on immunization was restricted to whether child received particular vaccine or not, and due to this data constraint, the study was unable to explore the specific reasons behind the dropout. Furthermore, the study did not differentiate between supply-side and demand-side determinants of the program, which could be useful in understanding the specific factors contributing to the dropout rate. While the study highlighted the association between the type of vaccine and the dropout rate, it did not identify the specific factors contributing to the higher dropout rate for OPV. Further research that explores the specific reasons behind the dropout and distinguishes between supply-side and demand-side factors could provide more nuanced insights into how to improve immunization coverage and reduce dropout rates.

## 5. Conclusions

The findings of this paper demonstrate that the dropout rate has reduced over the period of time. However, immunization dropout still poses a significant challenge in terms of ensuring that children receive the full protection provided by vaccines in India. Understanding the factors that contribute to immunization dropout and developing strategies to address these factors is essential for promoting public health, as well as for the country to achieve the target of full immunization coverage. Though the government and donor-funded programs have been consistently making efforts to improve childhood immunization, especially in the states of northern and central India, focus on the northeastern region has been limited. Continued efforts to improve access to vaccines, address vaccine hesitancy, and promote awareness about the benefits of vaccination are essential for reducing dropout and achieving the target of 90% full immunization coverage in a defined time frame.

## Figures and Tables

**Table 1:** The schedule of vaccination in India.

Schedule	Vaccinations
At birth	Bacillus Calmette Guerin (BCG), Hepatitis B-Birth Dose, Oral Polio vaccine (OPV)-0
At 6 weeks	OPV-1, DPT/Pentavalent-1, Rotavirus vaccine (RVV)-1
At 10 weeks	OPV-2, DPT/Pentavalent-2, RVV-2
At 14 weeks	OPV-3, DPT/Pentavalent-3, RVV-3
At 9 months	Measles/MR1

**Table 2:** Sample characteristics of the study population.

	NFHS-4		NFHS-5	
	%	*N*	%	*N*
**Mother’s Age**				
15–19 years	3.9	1718	3.6	1353
20–29 years	75.6	35,822	75.3	31,899
30 and above years	20.5	11,388	21.2	10,184
**Place of residence**				
Urban	28.4	11,700	26.9	8879
Rural	71.6	37,228	73.1	34,557
**Mother’s Education Level**				
No education	27.6	13,974	19.0	8475
Primary education	13.9	6985	11.3	5171
Secondary education	46.9	22,876	52.5	23,209
Higher education	11.6	5093	17.2	6581
Religion				
Hindu	78.3	35,172	79.6	32,212
Muslim	16.9	7822	16.1	6106
Others	4.8	5934	4.3	5118
**Wealth Index**				
Poorest	24.6	12,497	23.9	11,386
Poorer	21.6	11,306	21.4	10,073
Middle	20.2	9946	19.9	8518
Richer	18.7	8366	18.8	7446
Richest	14.9	6813	16.1	6013
**Birth Order**				
First	38.0	18,045	39.7	16,941
Second	33.2	15,558	34.3	14,463
Third	15.3	7793	14.9	6654
Fourth and above	13.5	7532	11.1	5378
**Sex of the Child**				
Male	51.9	25,431	52.0	22,537
Female	48.1	23,497	48.0	20,899
**Antenatal visits**				
No visits	16.3	7980	5.9	2559
1–3 visits	32.2	16,259	34.7	14,371
4 and above visits	51.5	21,970	59.4	23,887
**Place of delivery**				
Home	17.8	10,384	9.3	4937
Public health facilities	54.6	27,821	63.2	28,623
Private health facilities	27.5	10,604	27.6	9786
**Place of vaccination**				
Public health facilities	92.7	42,423	85.6	11,869
Private health facilities	7.3	2354	14.4	1175

**Table 3:** Percentage distribution of dropout rates by background characteristics in NFHS-4 and NFHS-5.

	NFHS-4		NFHS-5		NFHS-4		NFHS-5		NFHS-4		NFHS-5	
	DO BCG-MCV1		DO BCG-MCV1		DO OPV1-OPV3		DO OPV1-OPV3		DO DPT1-DPT3		DO DPT1-DPT3	
	%	*N*	%	*N*	%	*N*	%	*N*	%	*N*	%	*N*
**Mother’s Age**												
15–19 years	13.4	248	9.5	141	19.3	350	15.9	193	12.9	233	8.0	125
20–29 years	12.4	4418	8.5	2553	19.4	6453	12.8	3604	12.0	4152	7.1	2129
30 and above years	14.3	1502	9.1	842	20.9	2063	12.9	1142	13.7	1347	8.1	684
**Place of Residence**												
Urban	11.5	1370	8.8	724	20.0	2008	13.7	1043	11.2	1269	7.1	558
Rural	13.3	4798	8.5	2812	19.5	6858	12.6	3896	12.9	4463	7.5	2380
**Mother’s Education Level**												
No education	18.4	2286	13.3	1002	24.3	2992	17.3	1232	17.5	2084	10.3	779
Primary education	14.2	1000	9.7	494	21.0	1346	13.6	645	13.2	885	8.7	408
Secondary education	10.7	2495	7.5	1694	17.5	3777	11.5	2430	10.6	2364	6.5	1435
Higher education	7.2	387	6.5	346	16.5	751	12.1	632	8.0	399	6.3	316
**Religion**												
Hindu	12.0	4103	7.9	2271	19.5	6363	12.5	3467	12.0	4023	7.0	1989
Muslim	17.1	1242	12.7	719	21.8	1490	15.3	814	15.2	1045	9.6	543
Others	11.4	823	7.0	546	14.9	1013	10.9	658	9.7	664	5.9	406
**Wealth Index**												
Poorest	17.1	1969	11.5	1237	23.4	2708	16.1	1607	16.7	1822	9.4	1024
Poorer	14.5	1628	9.1	887	20.4	2226	13.0	1160	13.3	1469	8.1	739
Middle	12.1	1160	7.2	582	18.9	1656	11.3	874	11.7	1062	5.9	475
Richer	10.5	866	7.7	488	17.8	1311	11.6	710	10.2	853	7.3	419
Richest	7.6	545	6.6	342	16.3	965	11.7	588	8.3	526	5.6	281
**Birth Order**												
First	10.5	1936	6.8	1121	17.2	2990	11.6	1719	10.2	1833	5.8	938
Second	11.9	1857	8.4	1114	19.0	2770	12.6	1599	11.5	1753	7.5	961
Third	15.5	1134	10.5	641	23.4	1553	14.9	847	15.0	1033	8.6	523
Fourth and above	18.9	1241	13.7	660	24.6	1553	16.4	774	18.5	1113	11.0	516
**Sex of the Child**												
Male	12.2	3129	8.5	1795	20.3	4690	12.8	2503	12.7	3018	7.3	1503
Female	13.4	3039	8.8	1741	18.9	4176	13.1	2436	12.1	2714	7.5	1435
**Antenatal visits**												
No visits	21.4	1444	14.5	360	26.3	1768	18.8	421	20.7	1323	11.5	270
1–3 visits	14.8	2325	10.9	1399	22.0	3297	15.7	1920	14.5	2189	9.3	1203
4 or above visits	9.2	2066	6.8	1556	16.2	3278	10.6	2280	8.8	1890	6.0	1292
**Place of delivery**												
Home	18.6	1703	13.8	662	24.0	2205	18.9	821	19.1	1630	12.2	548
Public health facilities	12.4	3360	8.3	2192	18.7	4844	12.0	2998	11.5	2988	7.0	1821
Private health facilities	10.2	1084	7.8	676	19.0	1792	13.1	1111	10.5	1091	6.9	564
**Place of vaccination**												
Public health facilities	12.6	5632	9.9	1151	19.4	8181	12.9	1409	12.2	5247	7.2	819
Private health facilities	12.0	319	13.8	151	23.6	492	20.0	213	12.0	289	10.5	92

**Table 4:** Factors affecting dropout rates in NFHS-4 and NFHS-5 using a logistic regression model.

	NFHS-4		NFHS-5		NFHS-4		NFHS-5		NFHS-4		NFHS-5	
	DO BCG-MCV1		DO BCG-MCV1		DO OPV1-OPV3		DO OPV1-OPV3		DO DPT1-DPT3		DO DPT1-DPT3	
	Odds Ratio	95% CI	Odds Ratio	95% CI	Odds Ratio	95% CI	Odds Ratio	95% CI	Odds Ratio	95% CI	Odds Ratio	95% CI
**Mother’s Age**												
15–19 years^®^												
20–29 years	0.81 ***	(0.69–0.94)	0.56 ***	(0.4–0.78)	0.85 **	(0.74–0.97)	0.70**	(0.5–0.97)	0.79 ***	(0.66–0.91)	0.53 ***	(0.36–0.77)
30 and above years	0.73 ***	(0.61–0.86)	0.42 ***	(0.29–0.61)	0.80 ***	(0.69–0.92)	0.56 ***	(0.39–0.79)	0.70 ***	(0.58–0.83)	0.41 ***	(0.27–0.62)
**Place of Residence**												
Urban^®^												
Rural	0.81 ***	(0.75–0.88)	0.74 ***	(0.64–0.86)	0.87 ***	(0.82–0.93)	0.78 ***	(0.68–0.89)	0.83 ***	(0.76–0.9)	0.85 *	(0.72–1.02)
**Mother’s Education Level**												
No education^®^												
Primary education	0.92 *	(0.84–1.01)	0.85	(0.68–1.06)	0.92 **	(0.85–0.99)	0.87	(0.7–1.09)	0.88 ***	(0.8–0.97)	0.95	(0.72–1.24)
Secondary education	0.77 ***	(0.72–0.84)	0.69 ***	(0.58–0.82)	0.83 ***	(0.78–0.89)	0.82 **	(0.69–0.97)	0.79 ***	(0.73–0.86)	0.85	(0.69–1.06)
Higher education	0.63 ***	(0.54–0.72)	0.56 ***	(0.44–0.72)	0.82 ***	(0.73–0.92)	0.74 ***	(0.59–0.92)	0.70 ***	(0.6–0.8)	0.77 *	(0.57–1.03)
**Religion**												
Hindu^®^												
Muslim	1.53 ***	(1.42–1.65)	1.45 ***	(1.23–1.71)	1.13 ***	(1.05–1.21)	1.13	(0.96–1.32)	1.27 ***	(1.17–1.38)	1.39 ***	(1.14–1.68)
Others	1.49 ***	(1.36–1.62)	1.25 ***	(1.07–1.46)	1.03	(0.95–1.12)	1.19 **	(1.03–1.37)	1.16 ***	(1.05–1.28)	1.16	(0.97–1.39)
**Wealth Index**												
Poorest^®^												
Poorer	1.01	(0.93–1.09)	0.78 ***	(0.65–0.93)	0.96	(0.9–1.03)	0.86	(0.72–1.04)	0.97	(0.89–1.05)	0.83 *	(0.67–1.03)
Middle	0.88 ***	(0.8–0.96)	0.59 ***	(0.48–0.72)	0.81 ***	(0.75–0.88)	0.72 ***	(0.59–0.87)	0.86 ***	(0.78–0.94)	0.63 ***	(0.5–0.8)
Richer	0.78 ***	(0.7–0.87)	0.55 ***	(0.44–0.69)	0.76 ***	(0.7–0.84)	0.62 ***	(0.5–0.77)	0.84 ***	(0.75–0.93)	0.59 ***	(0.46–0.77)
Richest	0.61 ***	(0.53–0.69)	0.44 ***	(0.34–0.57)	0.65 ***	(0.58–0.73)	0.69 ***	(0.55–0.87)	0.60 ***	(0.52–0.69)	0.55 ***	(0.41–0.74)
**Birth Order**												
First^®^												
Second	1.06	(0.98–1.14)	1.16 **	(1–1.35)	1.05	(0.99–1.12)	1.11	(0.97–1.26)	1.07	(0.99–1.15)	1.15	(0.96–1.37)
Third	1.22 ***	(1.12–1.34)	1.50 ***	(1.23–1.82)	1.13 ***	(1.05–1.22)	1.37 ***	(1.14–1.64)	1.16 ***	(1.06–1.27)	1.40 ***	(1.11–1.75)
Fourth and above	1.26 ***	(1.14–1.39)	1.88 ***	(1.51–2.36)	1.14 ***	(1.04–1.24)	1.48 ***	(1.19–1.84)	1.24 ***	(1.11–1.38)	1.73 ***	(1.33–2.26)
**Sex of the Child**												
Male^®^												
Female	1.04	(0.98–1.1)	0.95	(0.84–1.07)	0.94 ***	(0.89–0.99)	1.02	(0.91–1.14)	0.94 **	(0.89–1)	1.02	(0.88–1.17)
**Antenatal visits**												
No visits^®^												
1–3 visits	0.67 ***	(0.62–0.73)	0.89	(0.71–1.12)	0.80 ***	(0.74–0.86)	0.95	(0.76–1.19)	0.69 ***	(0.63–0.74)	0.91	(0.69–1.19)
4 or above visits	0.47 ***	(0.43–0.51)	0.55 ***	(0.44–0.69)	0.57 ***	(0.53–0.62)	0.62 ***	(0.5–0.77)	0.45 ***	(0.41–0.49)	0.60 ***	(0.46–0.78)
**Place of delivery**												
Home^®^												
Public health facilities	0.81 ***	(0.75–0.87)	0.81 **	(0.66–1)	0.83 ***	(0.78–0.89)	0.72 ***	(0.59–0.88)	0.70 ***	(0.65–0.75)	0.66 ***	(0.52–0.83)
Private health facilities	0.78 ***	(0.71–0.87)	0.8	(0.62–1.04)	0.89 ***	(0.82–0.97)	0.81 *	(0.64–1.02)	0.78 ***	(0.71–0.87)	0.71 **	(0.53–0.95)
**Place of vaccination**												
Public health facilities^®^												
Private health facilities	1.69 ***	(1.46–1.95)	2.24 ***	(1.78–2.81)	1.53 ***	(1.36–1.73)	2.05 ***	(1.69–2.49)	1.58 ***	(1.36–1.83)	1.64 ***	(1.25–2.15)

Significance: * *p* < 0.05; ** *p* < 0.01; *** *p* < 0.001.

**Table 5:** Fairlie decomposition results of gaps in dropout rates in the NFHS-4 and NFHS-5 surveys.

	DO BCG-MCV1		DO OPV1-OPV3		DO DPT1-DPT3	
Covariates	Coeff.	SE	Coeff.	SE	Coeff.	SE
Mother’s Age	0.0006	0.0001	0.0008	0.0002	0.0006	0.0001
Place of Residence	−0.0031	0.0006	−0.0027	0.0007	−0.0028	0.0006
Mother’s Education Level	0.0081	0.0009	0.0068	0.0012	0.0068	0.0010
Religion	−0.0089	0.0008	−0.0014	0.0006	−0.0036	0.0007
Wealth Index	0.0056	0.0008	0.0069	0.0009	0.0043	0.0008
Birth Order	0.0022	0.0004	0.0016	0.0004	0.0016	0.0004
Sex of the Child	0.0000	0.0000	0.0000	0.0000	0.0000	0.0000
Antenatal Visits	0.0142	0.0008	0.0134	0.0008	0.0137	0.0008
Place of Delivery	0.0024	0.0005	0.0015	0.0005	0.0025	0.0005
Place of Vaccination	−0.001	0.0002	−0.0019	0.0002	−0.001	0.0002
Mean prediction in NFHS-4	0.137		0.200		0.129	
Mean prediction in NFHS-5	0.101		0.132		0.073	
Total Gap	0.036		0.069		0.057	
Explained Gap	0.020		0.025		0.022	
Explained Gap (%)	55.9		36.5		39.2	
Sample size	53,187		52,272		51,897	

## Data Availability

**The data are available in the public domain and can be downloaded upon request. accessed on 29 December 2022**, https://dhsprogram.com/data/available-datasets.cfm.
